# Genetic Variation in the TNF Gene Is Associated with Susceptibility to Severe Sepsis, but Not with Mortality

**DOI:** 10.1371/journal.pone.0046113

**Published:** 2012-09-27

**Authors:** Zhenju Song, Yuanlin Song, Jun Yin, Yao Shen, Chenling Yao, Zhan Sun, Jinjun Jiang, Duming Zhu, Yong Zhang, Qinjun Shen, Lei Gao, Chaoyang Tong, Chunxue Bai

**Affiliations:** 1 Department of Emergency Medicine, Zhongshan Hospital, Fudan University, Shanghai, People's Republic of China; 2 Department of Pulmonary Medicine, Zhongshan Hospital, Fudan University, Shanghai, People's Republic of China; 3 Department of Anesthesiology, Zhongshan Hospital, Fudan University, Shanghai, People's Republic of China; University of Washington, United States of America

## Abstract

**Background:**

Tumor necrosis factor (TNF) and TNF receptor superfamily (TNFR)-mediated immune response play an essential role in the pathogenesis of severe sepsis. Studies examining associations of *TNF* and *lymphotoxin-α* (*LTA*) single nucleotide polymorphisms (SNPs) with severe sepsis have produced conflicting results. The objective of this study was to investigate whether genetic variation in *TNF*, *LTA*, *TNFRSF1A* and *TNFRSF1B* was associated with susceptibility to or death from severe sepsis in Chinese Han population.

**Methodology/Principal Findings:**

Ten SNPs in *TNF*, *LTA*, *TNFRSF1A* and *TNFRSF1B* were genotyped in samples of patients with severe sepsis (n = 432), sepsis (n = 384) and healthy controls (n = 624). Our results showed that rs1800629, a SNP in the promoter region of *TNF*, was significantly associated with risk for severe sepsis. The minor allele frequency of rs1800629 was significantly higher in severe sepsis patients than that in both healthy controls (P_adj_ = 0.00046, odds ratio (OR)_adj_ = 1.92) and sepsis patients (P_adj_ = 0.002, OR_adj_ = 1.56). Further, we investigated the correlation between rs1800629 genotypes and TNF-α concentrations in peripheral blood mononuclear cells (PBMCs) of healthy volunteers exposed to lipopolysaccharides (LPS) *ex vivo*, and the association between rs1800629 and TNF-α serum levels in severe sepsis patients. After exposure to LPS, the TNF-α concentration in culture supernatants of PBMCs was significantly higher in the subjects with AA+AG genotypes than that with GG genotype (P = 0.007). Moreover, in patients with severe sepsis, individuals with AA+AG genotypes had significantly higher TNF-α serum concentrations than those with GG genotype (P_adj_ = 0.02). However, there were no significant associations between SNPs in the four candidate genes and 30 day mortality for patients with severe sepsis.

**Conclusions/Significance:**

Our findings suggested that the functional TNF gene SNP rs1800629 was strongly associated with susceptibility to severe sepsis, but not with lethality in Chinese Han population.

## Introduction

Sepsis is an infection-initiated and inflammation-induced syndrome. Despite progress in the development of antibiotics and other supportive care therapies, severe sepsis remains an unconquered challenge for the clinicians with an unacceptable high mortality rate of 30%–50% [1]. The response to infection is diverse among different individuals. Given the same therapies, most sepsis patients will recover and do well, while a small, but significant portion, will develop severe sepsis and multiple organ system failure, refractory hypotension and death [2], [3]. Currently, more and more evidence showed that genetic factors played an important role in the development and severity of sepsis [4], [5], [6], [7], [8], [9], [10], [11]. Common sequence variants within genes involved in pro-inflammatory response have received particular attention [12], [13].

Although the pathogenesis of sepsis remains incompletely understood, an excessive pro-inflammatory response has been established as a fundamental component of severe sepsis [14]. The proinflammatory cytokine TNF-α is an essential component in the host immune response to infection and has been widely reported to be an important mediator in severe sepsis and septic shock. High circulating levels of TNF-α were correlated with poor outcomes in sepsis patients [15]. TNF-α and lymphotoxin-α (LT-α) share the same receptors as well as many biological activities, and they are central mediators of immune responses [16]. TNF-α and LT-α are encoded by adjacent gene loci in the central or class III region of the human major histocompatibility complex (MHC), between the HLA class I and II genes on the short arm of chromosome 6 [17]. Several SNPs within the promoter region of *TNF* (−238, −308, −857, −863, −1031) and the first intron of *LTA* (+252) were thought to influence TNF-α and LT-α production, and have therefore been identified as candidate variants that might influence susceptibility to and/or outcomes from severe sepsis and infectious diseases [18], [19], [20], [21], [22], [23], [24], [25]. In particular, rs1800629 (*TNF* −308) and rs909253 (*LTA* +252) have been the focus of many investigations on sepsis. Although several studies have identified associations for rs1800629 and rs909253 with sepsis risk or outcomes [18], [19], [20], [23], [24], other studies have not replicated the associations [21], [26], [27], [28]. This inconsistency may be due to small samples size studied and ethnic differences [29].

TNF-α and LT-α exert their pleiotropic functions by activating intracellular signaling cascades via binding to two types of receptors, TNFR-1 (encoded by the TNFRSF1A gene) and TNFR-2 (encoded by the TNFRSF1B gene) [30]. TNFR1-deficient mice are resistant to endotoxic shock and have prolonged survival with less hypothermia [31]. TNFR2 influences the biological activity of TNF-α and LT-α both in a membrane-bound and a soluble form. Membrane-bound TNFR2 facilitates activation of nuclear factor (NF)-κB and mitogen-activated protein kinase signaling cascades upon binding with TNF-α and LT-α, whereas soluble TNFR2 is capable of binding and inactivating circulating TNF-α and LT-α [16], [30]. Moreover, animal studies showed that TNFR2 mediated protective effects in the development of severe sepsis [31]. Recent studies proposed that genetic variation in *TNFRSF1A* and *TNFRSF1B* was associated with susceptibility to inflammatory and autoimmune diseases, such as tuberculosis, systemic lupus erythematosus, rheumatoid arthritis and Crohn's disease [32], [33], [34], [35]. However, to date, only one study investigated the role of *TNFRSF1A* and *TNFRSF1B* polymorphisms in sepsis susceptibility and mortality [36].

Considering the important role of TNF-α, LT-α, TNFR1 and TNFR2 in the pathogenesis of severe sepsis, we hypothesized that genetic variation in *TNF*, *LTA*, *TNFRSF1A* and *TNFRSF1B* might be associated with susceptibility to and outcomes from severe sepsis in Chinese Han population. To test this hypothesis, we conducted a relatively large-scale case-control study enrolling 432 severe sepsis patients, 384 sepsis patients and 624 healthy individuals to investigate the association of genetic variants in *TNF*, *LTA*, *TNFRSF1A* and *TNFRSF1B* with severe sepsis susceptibility and prognosis in Chinese Han population. Furthermore, we investigated the association between the genotypes of the TNF gene SNP rs1800629 and TNF-α concentration in culture supernatants of LPS simulated PBMCs obtained from healthy donors and in serum from severe sepsis patients.

## Results

### Characteristics of Study Subjects

A total of 432 severe sepsis patients, 384 sepsis patients and 624 health volunteers were enrolled in this case-control study. According to the mortality within 30 days, severe sepsis patients were divided into survivor and non-survivor groups. The baseline characteristics and clinical data of all subjects are shown in Table 1. The average age and proportion of male among the severe sepsis, sepsis and healthy control groups did not show significant difference. The primary source of infection in severe sepsis patients was the lungs (69.9%), followed by abdomen (21.8%), blood stream (3.5%), urinary tract (2.5%) and others (2.3%). The overall 30-day mortality rate of severe sepsis patients was 36.1%. The mean APACHE II and SOFA score in non-survivor group was higher than that in survivor group (P<0.05).

**Table 1:** Demographic and clinical characteristics of the study subjects.

	Healthy controls	Sepsis patients	Severe sepsis patients	P^1^ value	Survivor	Nonsurvivor	P^2^ value
Number	624	384	432	N.A	276	156	N.A
Age	68.5±9.3	62.1±10.8	65.1±11.8	0.12	63.4±10.8	68.1±14.2	0.09
Sex(Male/Female)	363/261	220/164	256/176	0.57	163/113	93/63	0.91
APACHE II score	N.A	10.2±3.2	18.6±4.9	0.008	14.8±2.9	25.3±7.2	0.013
Length of ICU stay (d)	N.A	8.6±2.3	15.4±8.6	0.006	14.6±7.2	16.8±8.9	0.02
Diabetes	N.A	35 (9.1%)	46 (10.6%)	0.47	30 (10.8%)	16 (10.2%)	0.84
Chronic liver disease	N.A	9 (2.3%)	15 (3.5%)	0.34	8 (2.9%)	7 (4.5%)	0.39
Chronic renal failure	N.A	14 (3.6%)	18 (4.2%)	0.70	12 (4.3%)	6 (3.8%)	0.80
Congestive heart failure	N.A	22 (5.7%)	28 (6.5%)	0.66	18 (6.5%)	10 (6.4%)	0.96
Chronic pulmonary disease	N.A	28 (7.3%)	37 (8.6%)	0.50	22 (8.0%)	15 (9.6%)	0.56
SOFA score	N.A	1.4±0.3	8.2±1.6	<0.001	6.9±1.8	10.5±1.9	0.011
Failing organs (score >2 in SOFA scale)							
Respiratory	N.A	N.A	293 (67.8%)	N.A	156 (56.5%)	137 (87.8%)	<0.001
Cardiovascular	N.A	N.A	252 (58.3%)	N.A	141 (51.1%)	111 (71.2%)	<0.001
Kidney	N.A	N.A	112 (25.9%)	N.A	46 (16.7%)	66 (42.3%)	<0.001
Neurologic	N.A	N.A	59 (13.7%)	N.A	29 (10.5%)	30 (19.2%)	0.01
Liver	N.A	N.A	42 (9.7%)	N.A	18 (6.5%)	24 (15.4%)	0.003
Hematologic	N.A	N.A	35 (8.1%)	N.A	14 (5.1%)	21 (13.5%)	0.002
Infection Insult							
Lung	N.A	261 (68.0%)	302 (69.9%)	0.55	198 (71.7%)	104 (66.7%)	0.27
Abdomen	N.A	92 (24.0%)	94 (21.8%)	0.46	57 (20.7%)	37 (23.7%)	0.46
Bloodstream	N.A	11 (2.9%)	15 (3.5%)	0.62	7 (2.5%)	8 (5.1%)	0.16
UTI	N.A	9 (2.3%)	11 (2.5%)	0.85	8 (2.9%)	3 (1.9%)	0.52
Others	N.A	11 (2.9%)	10 (2.3%)	0.62	6 (2.2%)	4 (2.6%)	0.80
Microbiology positive	N.A	153 (39.8%)	174 (40.3%)	0.90	101 (36.6%)	73 (46.8%)	0.04
Gram positive	N.A	63 (41.2%)	66 (37.9%)	0.55	41 (40.6%)	25 (34.2%)	0.39
Gram negative	N.A	73 (47.7%)	72 (41.4%)	0.25	43 (42.6%)	29 (39.7%)	0.71
Fungi	N.A	7 (1.8%)	17 (9.8%)	0.07	9 (8.9%)	8 (11.0%)	0.65
Mixed	N.A	10 (6.5%)	19 (10.9%)	0.16	8 (7.9%)	11 (15.1%)	0.14
Microbiology unknown	N.A	231 (60.2%)	258 (59.7%)	0.90	175 (63.4%)	83 (53.2%)	0.04

N.A, not applicable; APACHE, acute physiology and chronic health evaluation; SOFA, sequential organ failure assessment. P^1^, sepsis group vs severe sepsis group. P^2^, survivor group vs non-survivor group.

### Association Analyses of *TNF*, *LTA*, *TNFRSF1A* and *TNFRSF1B* SNPs with Susceptibility to Severe Sepsis

The genotyping success rates of all tested SNPs ranged from 95% to 99% and none of the ten SNPs diverged significantly from Hardy-Weinberg equilibrium (P>0.05) (Table 2). The allele and genotype distributions of all tested SNPs in severe sepsis patients, sepsis patients and healthy controls are listed in Table 3. Our results showed that rs1800629 (−308G/A), located in the promoter region of *TNF*, was associated with significantly increased risk for severe sepsis. The frequency of rs1800629A in severe sepsis patients was significantly higher than that in both the healthy control subjects (P = 0.00028, OR = 2.08) and the sepsis patients (P = 0.00035, OR = 2.39), and the difference remained significant after Bonferroni correction. Moreover, in multivariate analyses after adjustment for covariates, rs1800629A was still significantly associated with the development of severe sepsis when compared with healthy control group (P_adj_ = 0.00046, OR_adj_ = 1.92) and sepsis group (P_adj_ = 0.002, OR_adj_ = 1.56). The genotype distribution of rs1800629 in the severe sepsis group was also significantly different from that in the healthy control group (P_adj_ = 0.003) and the sepsis group (P_adj_ = 0.004), and the significance remained after Bonferroni correction. However, the difference of the allele and genotype frequencies of rs1800629 between subjects with sepsis and healthy controls were not statistically significant (P>0.05). When we analyzed the allele and genotype distributions of the other nine SNPs (rs361525, rs1799724, rs1799964, rs767455, rs4149570, rs1061622, rs3397, rs1800630 and rs909253), no significant difference was found between the severe sepsis, sepsis and healthy control groups (Table 3).

**Table 2:** Characteristics of all tested SNPs in *TNF*, *LTA*, *TNFRSF1A* and *TNFRSF1B*.

Gene	SNP	Location	Major/minor allele	HWE P value
TNF	rs361525 (−238)	promoter	C/T	0.46
	rs1800629 (−308)	promoter	G/A	0.18
	rs1799724 (−857)	promoter	G/A	0.67
	rs1800630 (−863)	promoter	C/A	0.39
	rs1799964 (−1031)	promoter	T/C	0.49
LTA (TNF-β)	rs909253 (+252)	intron	A/G	0.58
TNFRSF1A	rs767455 (A36G)	exon	A/G	0.22
	rs4149570 (G609T)	5′UTR	G/T	0.72
TNFRSF1B	rs1061622 (Met196Arg)	exon	T/G	0.31
	rs3397	3′UTR	G/A	0.44

SNP, single nucleotide polymorphism; HWE, Hardy-Weinberg equilibrium; UTR, untranslated region.

**Table 3:** Association analysis of SNPs in *TNF*, *LTA*, *TNFRSF1A* and *TNFRSF1B* between case and control groups.

Gene	Healthy	Sepsis	Severe sepsis	Allelic Comparison				Genotypic Comparison	
SNP	controls	patients	patients	P^1^_adj_	OR^1^_adj_ 95% CI	P^2^_adj_	OR^2^_adj_ 95% CI	P^1^_adj_	P^2^_adj_
TNF									
rs1800629				0.00046	1.92 (1.26–2.92)	0.002	1.56 (1.23–2.76)	0.003	0.004
GG	560 (93.3%)	352 (93.9%)	369 (86.4%)						
GA	38 (6.3%)	23 (6.1%)	56 (13.1%)						
AA	2 (0.3%)	0 (0%)	2 (0.47%)						
G	1158 (96.5%)	727 (96.9%)	794 (93%)						
A	42 (3.5%)	23 (3.1%)	60 (7%)						
rs361525				0.22	1.09 (0.98–1.87)	0.88	1.02 (0.84–1.42)	0.21	0.89
CC	550 (92%)	333 (88.8%)	382 (89.3%)						
CT	48 (8%)	42 (11.2%)	46 (10.7%)						
C	1148 (96%)	708 (94.4%)	810 (94.6%)						
T	48 (4%)	42 (5.6%)	46 (5.4%)						
rs1799724				0.21	1.18 (0.98–2.01)	0.52	1.06 (0.86–1.46)	0.12	0.85
GG	444 (73.6%)	263 (70.3%)	285 (67.9%)						
GA	149 (24.7%)	107 (28.6%)	130 (31%)						
AA	10 (1.7%)	4 (1.1%)	5 (1.2%)						
G	1037 (86%)	633 (84.6%)	700 (83.3%)						
A	169 (14%)	115 (15.4%)	140 (16.7%)						
rs1799964				0.62	1.02 (0.92–1.42)	0.56	1.12 (0.84–1.46)	0.87	0.52
TT	384 (64.4%)	234 (62.2%)	265 (62.6%)						
TC	188 (31.5%)	130 (34.6%)	140 (33.1%)						
CC	24 (4%)	12 (3.2%)	18 (4.3%)						
T	956 (80.2%)	598 (79.5%)	670 (79.2%)						
C	236 (19.8%)	154 (20.5%)	176 (20.8%)						
rs1800630				0.32	1.09 (0.81–1.40)	0.38	1.18 (0.72–1.43)	0.12	0.42
CC	412 (69%)	262 (70.6%)	275 (66.3%)						
AC	179 (30%)	102 (27.5%)	128 (30.8%)						
AA	6 (1%)	7 (1.9%)	12 (2.9%)						
C	1003 (84%)	626 (84.4%)	678 (81.7%)						
A	191 (16%)	116 (15.6%)	152 (18.3%)						
LTA									
rs909253				0.18	0.89 (0.79–1.21)	0.36	0.82 (0.58–1.26)	0.29	0.14
AA	178 (29.7%)	103 (27.8%)	140 (33.6%)						
AG	266 (44.4%)	181 (48.9%)	181 (43.5%)						
GG	155 (25.9%)	86 (23.2%)	95 (22.8%)						
A	622 (51.9%)	387 (52.3%)	461 (55.4%)						
G	576 (48.1%)	353 (47.7%)	371 (44.6%)						
TNFRSF1A									
rs767455				0.16	1.20 (0.82–1.68)	0.72	1.04 (0.76–1.23)	0.27	0.76
AA	462 (76.4%)	276 (73.4%)	302 (71.4%)						
GA	131 (21.7%)	90 (23.9%)	112 (26.5%)						
GG	12 (2.0%)	10 (2.7%)	9 (2.1%)						
A	1055 (87.2%)	642 (85.4%)	716 (84.6%)						
G	155 (12.8%)	110 (14.6%)	130 (15.4%)						
rs4149570				0.78	0.96 (0.67–1.09)	0.41	1.09 (0.82–1.34)	0.86	0.62
GG	178 (29.3%)	118 (31.6%)	123 (29.2%)						
GT	303 (49.9%)	192 (51.3%)	212 (50.4%)						
TT	126 (20.8%)	64 (17.1%)	86 (20.4%)						
G	659 (54.3%)	428 (57.2%)	458 (54.4%)						
T	555 (45.7%)	320 (42.8%)	384 (45.6%)						
TNFRSF1B									
rs1061622				0.32	0.92 (0.87–1.28)	0.32	1.02 (0.91–1.46)	0.35	0.38
TT	398 (65.9%)	262 (69.7%)	297 (70.5%)						
TG	190 (31.5%)	102 (27.1%)	116 (27.6%)						
GG	16 (2.6%)	12 (3.2%)	8 (1.9%)						
T	986 (81.6%)	626 (83.2%)	710 (84.3%)						
G	222 (18.4%)	126 (16.8%)	132 (15.7%)						
rs3397				0.48	1.04 (0.92–1.41)	0.17	1.28 (0.94–1.68)	0.30	0.32
GG	255 (41.9%)	156 (41.8%)	158 (37.6%)						
AG	304 (49.9%)	194 (52%)	232 (55.2%)						
AA	50 (8.2%)	23 (6.2%)	30 (7.2%)						
G	814 (66.8%)	506 (67.8%)	548 (65.2%)						
A	404 (33.2%)	240 (32.2%)	292 (34.8%)						

SNP, single nucleotide polymorphism; OR, odds ratio; CI, confidence interval. P_adj_ and OR_adj_ came from multivariate logistic regression. P^1^_adj_ and OR^1^_adj_, healthy control group vs severe sepsis group. P^2^_adj_ and OR^2^_adj_, sepsis group vs severe sepsis group. A P-value of <0.005 (0.05/10) was considered statistically significant after Bonferroni correction.

### Association Analyses of *TNF*, *LTA*, *TNFRSF1A* and *TNFRSF1B* SNPs with Severe Sepsis Outcomes

We next investigated the association between all tested SNPs and 30-day mortality. The overall 30-day mortality rate among severe sepsis patients was 36.1%. We compared the allele and genotype distributions of all tested SNPs between survivors and non survivors of severe sepsis patients. No association was observed between *TNF*, *LTA*, *TNFRSF1A and TNFRSF1B* variants and 30-day mortality in the severe sepsis cohort in either the unadjusted or adjusted models (Table 4).

**Table 4:** Association analysis of SNPs in *TNF*, *LTA*, *TNFRSF1A* and *TNFRSF1B* between survivors and non-survivors of severe sepsis patients.

Gene			Allelic Comparison		Genotypic Comparison
SNP	Nonsurvior	Survior	P_adj_	OR_adj_ 95% CI	P_adj_
TNF					
rs1800629			0.43	0.72 (0.58–1.32)	0.56
GG	137 (89%)	232 (85%)			
GA	17 (11%)	39 (14.3%)			
AA	0 (0%)	2 (0.7%)			
G	291 (94.5%)	503 (92.1%)			
A	17 (5.5%)	43 (7.9%)			
rs361525			0.68	1.15 (0.59–1.98)	0.62
CC	135 (87.7%)	247 (90.1%)			
CT	19 (12.3%)	27 (9.9%)			
C	289 (93.8%)	521 (95.1%)			
T	19 (6.2%)	27 (4.9%)			
rs1799724			0.21	0.76 (0.49–1.21)	0.12
GG	109 (71.7%)	176 (65.7%)			
GA	40 (26.3%)	90 (33.6%)			
AA	3 (2%)	2 (0.7%)			
G	258 (84.9%)	442 (82.5%)			
A	46 (15.1%)	94 (17.5%)			
rs1799964			0.68	1.04 (0.72–1.53)	0.78
TT	93 (60.8%)	172 (63.7%)			
TC	52 (34%)	88 (32.6%)			
CC	8 (5.2%)	10 (3.7%)			
T	238 (77.8%)	432 (80%)			
C	68 (22.2%)	108 (20%)			
rs1800630			0.42	1.18 (0.81–1.67)	0.76
CC	97 (63%)	178 (68.2%)			
AC	52 (33.7%)	76 (29.1%)			
AA	5 (3.3%)	7 (2.7%)			
C	246 (80%)	432 (82.8%)			
A	62 (20%)	90 (17.2%)			
LTA					
rs909253			0.26	0.82 (0.54–1.12)	0.38
AA	58 (37.4%)	82 (31.4%)			
AG	62 (40%)	119 (45.6%)			
GG	35 (22.6%)	60 (23%)			
A	178 (57.4%)	283 (54.2%)			
G	132 (42.6%)	239 (45.8%)			
TNFRSF1A					
rs767455			0.16	1.26 (0.87–1.86)	0.55
AA	106 (69.7%)	196 (72.3%)			
GA	41(27%)	71 (26.2%)			
GG	5 (3.3%)	4 (1.5%)			
A	253 (83.2%)	463 (85.4%)			
G	51 (16.8%)	79 (14.6%)			
rs4149570			0.65	1.15 (0.86–1.56)	0.26
GG	48 (31.4%)	75 (28%)			
GT	70 (45.6%)	142 (53%)			
TT	35 (23%)	51 (19%)			
G	166 (54.2%)	292 (54.5%)			
T	140 (45.8%)	244 (45.5%)			
TNFRSF1B					
rs1061622			0.64	1.18 (0.78–1.82)	0.66
TT	101 (67.3%)	196 (72.3%)			
TG	46 (30.7%)	70 (25.8%)			
GG	3 (2%)	5 (1.9%)			
T	248 (82.7%)	462 (85.2%)			
G	52 (17.3%)	80 (14.8%)			
rs3397			0.46	1.12 (0.82–1.61)	0.21
GG	52 (34.4%)	106 (39.4%)			
AG	90 (59.6%)	142 (52.8%)			
AA	9 (6%)	21 (7.8%)			
G	194 (64.2%)	354 (65.8%)			
A	108 (35.8%)	184 (34.2%)			

SNP, single nucleotide polymorphism; OR, odds ratio; CI, confidence interval. P_adj_ and OR_adj_ came from multivariate logistic regression. A P-value of <0.005 (0.05/10) was considered statistically significant after Bonferroni correction. The comparator group was survivors.

### Rs1800629 Genotypes were Associated with Elevated TNF-α Concentrations

**Figure 1:**
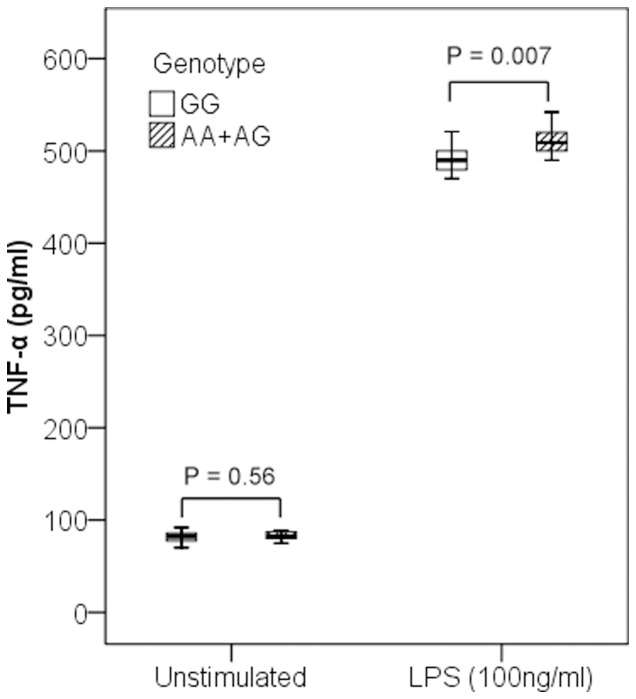
Association of TNF-α levels and rs1800629 genotypes in healthy volunteers. Concentrations of TNF-α in culture supernatants of PBMCs were expressed as the median, interquartile range and extremes. The TNF-α levels were significantly different between individuals with AA+GA and GG genotypes under the LPS-stimulated condition (P = 0.007). However, no significant difference was observed under the unstimulated condition (P = 0.56).

To determine whether rs1800629 genotypes influenced TNF-α production, we investigated TNF-α levels in culture supernatants of PBMCs obtained from 24 healthy volunteers. We observed a significant association between TNF-α levels and rs1800629 genotypes under the LPS-stimulated condition. AA+AG genotypes were associated with higher levels of TNF-α compared with GG genotype after LPS stimulation (P = 0.007) (Figure 1). However, no significant association was observed under the unstimulated condition.

**Figure 2:**
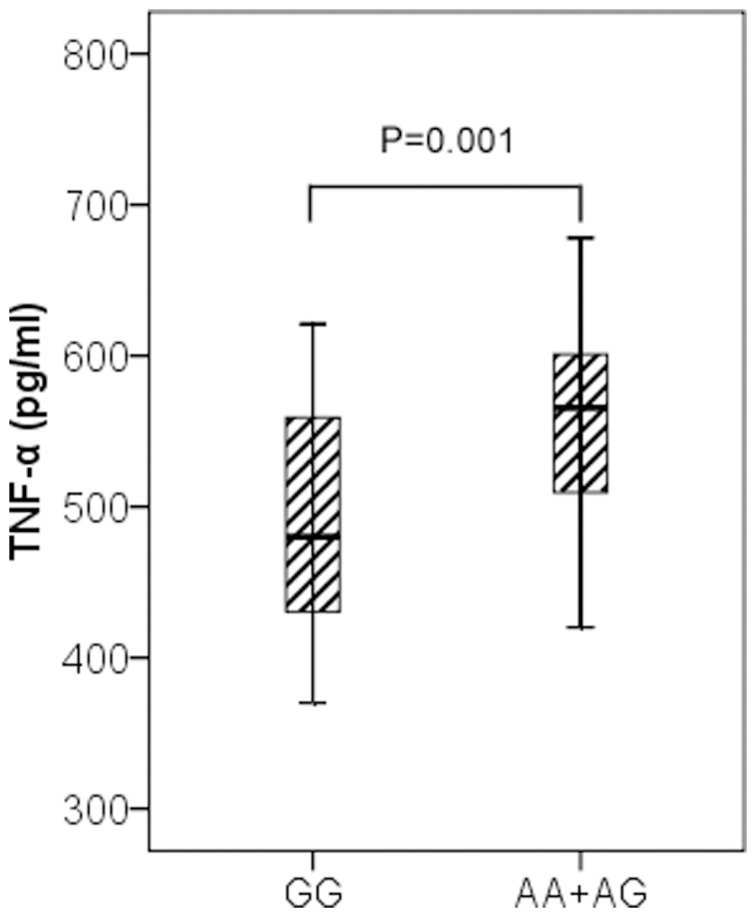
Association of TNF-α levels and rs1800629 genotypes in severe sepsis patients. TNF-α serum levels in severe sepsis patients were expressed as the median, interquartile range and extremes. The TNF-α levels were significantly different between individuals with AA+GA and GG genotypes (P = 0.001). The difference remained significant (P_adj_ = 0.02) after adjustment for age, gender and APACHE II scores in a linear regression model.

Furthermore, we measured TNF-α serum concentrations in 120 severe sepsis patients, including 104 patients with rs1800629GG genotype, 14 patients with GA genotype and 2 patients with AA genotype. Our results showed that rs1800629A allele was associated with higher TNF-α serum concentrations on the first day of severe sepsis. As shown in Figure 2, the serum concentration of TNF-α in severe sepsis patients with AA+AG genotypes was significantly higher than that of patients with GG genotype (550.4±73.6 pg/mL vs. 488.0±68.5 pg/mL, P = 0.001). To control confounding variables, we used the possible confounding factors (age, gender and APACHE II scores) as covariates in a linear regression model and found that the rs1800629 genotypes remained associated with TNF serum concentration (P_adj_ = 0.02).

## Discussion

Several genetic variants within genes involved in pro-inflammatory response have been associated with morbidity and mortality in patients with severe sepsis or septic shock, which is a complex and multifactorial syndrome [2]. TNF-α is an important pro-inflammatory cytokine involved in sepsis; several functional SNPs in *TNF* and *LTA* have been extensively studied in sepsis [29], [37]. However, results from previous studies were inconsistent. Discrepancies among previous studies may have resulted from differences in the populations studied, sepsis phenotype or imprecise definition of phenotype and limited sample size [38]. Considering these factors that might affect the results, we designed the present study with large samples size to achieve greater statistical power. Furthermore, all the samples were recruited from central Chinese Han population, thus the ethnic heterogeneity could be eliminated.

To our knowledge, this was the first relatively large-scale study investigating associations of genetic variants within *TNF*, *LTA*, *TNFRSF1A* and *TNFRSF1B* with severe sepsis in Chinese Han population. Our results provided evidence that rs1800629, a functional SNP in the promoter region of *TNF*, was significantly associated with susceptibility to severe sepsis in Chinese Han population. The association between rs1800629 and severe sepsis risk may be explained by its influence on the expression of TNF-α. In this study, we found that the risk allele (rs1800629A) is associated with increased TNF-α production in PBMCs from healthy subjects after stimulation with LPS. Moreover, we found that TNF-α serum levels in severe sepsis patients with AA+AG genotypes for rs1800629 were significantly higher than those in the individuals with GG genotype. Previous studies also showed that rs1800629A allele was associated with a six fold higher expression of both basal and induced *TNF* mRNA [39], [40]. Menges et al. found that the plasma TNF-α concentrations in patients with sepsis secondary to severe traumatic injury were significantly elevated in rs1800629A carriers on the first day after admission and for the following 14 days [41]. As TNF-α plays a pivotal role in the pathogenesis of severe sepsis in response to infection, it is reasonable to assume that patients with rs1800629A allele might produce a higher amount of TNF-α, and therefore become more susceptible to severe sepsis. In contrast to the *TNF* −308G/A polymorphism, the *LTA* +252A/G was not associated with the development of severe sepsis in our study. Our data showed that −308G/A of *TNF* and +252A/G of *LTA* were in weak linkage disequilibrium (LD) (D′ = 0.118) in Chinese Han population. The LD pattern is quite dissimilar to Caucasian population, which might result from the racial difference [24].

Rs1800629 was not associated with mortality among subjects with severe sepsis in our study. This was consistent with the study by Stuber et al., which demonstrated that the rs1800629 genotypes were not associated with poorer prognosis in severe sepsis. However, they did not find an association between rs1800629 genotypes and plasma TNF-α levels [26]. Recently, Teuffel et al. conducted a systematic review and meta-analysis, which also concluded that rs1800629 (*TNF* −308 AA/AG, TNF2) was associated with susceptibility to sepsis, but not with sepsis mortality [29].

Several studies have proposed that genetic variation in *TNFRSF1A* and *TNFRSF1B* was associated with susceptibility to inflammatory and autoimmune diseases, such as tuberculosis, systemic lupus erythematosus, rheumatoid arthritis and Crohn's disease [32], [33], [34], [35]. However, up to now, only one case control study investigated associations between *TNFRSF1A* and *TNFRSF1B* polymorphisms and sepsis susceptibility [36]. Four potentially functional SNPs in *TNFRSF1A* and *TNFRSF1B* were genotyped in our study. However, none showed association with susceptibility to or death from severe sepsis in Chinese Han population. Our findings were consistent with the results of Gordon et al. that five functional SNPs in *TNFRSF1A* and *TNFRSF1B* were not associated with susceptibility to or outcomes from sepsis in Caucasian population [36].

Potential limitations of this study should be addressed. First, although we knew that different pathogens had different impact on severity and outcomes of sepsis, we did not perform stratification analysis by different pathogens due to small number of cases with a definite microbiologic diagnosis. Second, we did not resequence these genes or select tag SNPs for genotyping. Instead, only ten potentially functional SNPs in *TNF*, *LTA*, *TNFRSF1A* and *TNFRSF1B* were included in our study, which was far from comprehensive. Indeed, these four genes are highly polymorphic. Therefore, it was possible that some important SNPs might be missed or the observed association might be due to other polymorphisms in LD with the studied ones. Additionally, assuming the prevalence of 0.01 for severe sepsis and using a significance level of 0.05, our study with 432 severe sepsis patients and 624 healthy controls had about 80% power to detect a 5% risk allele with an odds ratio of 1.63. Variant with an effect size smaller than this cannot convincingly be excluded based on these results. Therefore, our results cannot exclude variant associations with weaker effects between severe sepsis and the other three candidate genes (*LTA*, *TNFRSF1A*, *TNFRSF1B*). A more highly powered study involving thousands of subjects may yet exclude the role of these variants in severe sepsis susceptibility and outcomes.

In conclusion, our relatively large scale association study demonstrated that individuals with a functional variant in the promoter region of *TNF* may confer susceptibility to severe sepsis. However, common functional genetic variants in *TNF*, *LTA*, *TNFRSF1A* and *TNFRSF1B* were not associated with severe sepsis mortality in Chinese Han population.

## Materials and Methods

### Ethics Statement

This study was approved by the Ethics Study Board of Zhongshan Hospital, Fudan University, Shanghai, China (Record no: 2006-23). Written informed consent was obtained from patients or the next of kin, carers or guardians on the behalf of the participants before enrollment.

### Study Design and Enrollment

From May 2005 to March 2011, a total of 432 severe sepsis patients, 384 sepsis patients and 624 ethnic-matched healthy controls were enrolled in this study (Table 1). The severe sepsis patients were those admitted to the Emergency, Surgical and Respiratory ICU at Zhongshan Hospital. The sepsis patients were those admitted to Zhongshan Hospital, but did not develop severe sepsis during hospital stay. The sepsis patients were considered as at risk controls for severe sepsis. Of 384 sepsis patients, 174 patients overlapped with that from our previous study [42]. Another 210 sepsis patients were collected between May 2008 and March 2011, and these patients were not included in our previous study. Sepsis patients recruited in the current study included multi-trauma subjects and patients with a history of chronic heart, renal, liver or pulmonary failure, thus they spent a long time (more than 8 days on average) on ICU (Table 1). Sex- and age-matched controls were selected from healthy blood donors. To reduce the potential confounding from ethnic backgrounds, only central Han Chinese individuals were recruited in this study.

The diagnosis of sepsis was based on the criteria presented at the American College of Chest Physicians/Society of Critical Care Medicine Consensus Conference in 1992 [43]. Severe sepsis was defined as sepsis in combination with infection-induced acute organ dysfunction in at least one organ. Acute organ dysfunction was defined as Sequential Organ Failure Assessment (SOFA) scores more than 2 for the organ in question. Baseline characteristics (age, gender and previous health status), as well as clinical data including Acute Physiology and Chronic Health Evaluation II (APACHE II) and SOFA scores, source of infection, microbiology and ICU mortality were obtained after the patient met sepsis criteria. The APACHE II and SOFA scores were calculated in the first 24 hours after the diagnosis of sepsis and severe sepsis. All patients included in the protocol were followed up for 30 days or hospital discharge. When cultures were absent or negative, the source of infection was determined by two senior physicians. Exclusion criteria included age below 18 years, pregnancy, severe chronic respiratory disease, severe chronic liver disease (defined as a Child–Pugh score of >10), malignancy, using of high-dose immunosuppressive therapy and AIDS diagnosis. Questionnaires were obtained from all control subjects to document smoking status, and history of chronic illness or severe sepsis. Healthy controls were defined as individuals without any recent acute illness, chronic illness or history of sepsis and severe sepsis.

### SNPs Selection and Genotyping

Previous studies found that several functional SNPs in *TNF*, *LTA*, *TNFRSF1A* and *TNFRSF1B* were associated with inflammatory and autoimmune diseases. In our study, SNPs in *TNF*, *LTA*, and *TNFRSF1A* and *TNFRSF1B* were selected based on the following criteria: (1) location within the gene region (promoter, intron, exon, 3′UTR and 5′UTR); (2) association with inflammatory and autoimmune diseases such as sepsis, asthma, tuberculosis, systemic lupus erythematosus, rheumatoid arthritis and Crohn's disease in more than two studies. A total of ten SNPs were selected and genotyped in our study. Location and characterization of all selected SNPs were listed in Table 2.

Genomic DNA was extracted from whole blood with a FlexiGene DNA Kit (Qiagen, Hilden, Germany) in accordance with the protocol of the manufacturer. Six SNPs (rs1800629, rs1799724, rs361525, rs1800630, rs1799964 and rs909253) in *TNF* and *LTA* were selected and genotyped by direct sequencing. The sequencing reactions were performed using Applied Biosystems BigDye (version 3.1) chemistry (Applied Biosystem, Foster City, CA, USA), and the sequences were resolved using an ABI 3730 Genetic Analyzer. Analyses of the sequence traces were performed using the Staden package and were double scored by a second operator. The primers and PCR protocols used were shown in Table S1. Four SNPs in *TNFRSF1A* (rs767455, rs4149570) and *TNFRSF1B* (rs1061622, rs3397) were selected and genotyped on the GenomeLab SNPstream high-throughput 12-plex genotyping platform (Beckman Coulter, Fullerton, CA) following the manufacturer's instructions. The primers for PCR and single base extension were performed with Beckman Coulter Autoprimer software and shown in Table S2.

### Isolation and Stimulation of Cells from Healthy Subjects

To determine the associations between rs1800629 genotypes and TNF-α levels in PBMCs, we investigated 15 subjects with rs1800629GG genotype, 8 subjects with GA genotype and 1 subject with AA genotype. PBMCs were derived by using Ficoll gradient density centrifugation method. Isolated PBMCs were plated at a density of 1×10^6^ cells/ml in 24-well plates and cultured in RPMI 1640 medium with 10% FBS at 37 °C with 5% CO_2_. The cells were then incubated for 6 hours in presence or absence of 100 ng/ml Escherichia coli 0111:B4 LPS (Sigma, USA). After incubation, supernatants were harvested and stored at −80°C until use.

### Serum Collection and TNF-α Level Measurement

Blood samples (5 mL) were collected within 24 hours of meeting criteria for severe sepsis. Samples were centrifuged at 4°C for 10 min at 3200 rpm within 60 min after collection. Then the serum was stored at −80°C until use. TNF-α level was determined by human ELISA kit (R&D Systems, USA) according to the manufacturer's protocol.

### Statistical Analysis

The genotype data of cases and controls was analyzed for deviations from Hardy-Weinberg equilibrium by the Haploview v4.1 software [44]. The differences in allele and genotype distributions between severe sepsis and control groups were compared using χ^2^-test or Fisher's exact test when appropriate. The test for association with genotypes used the global genotype test in the 3×2 contingency table. Allele frequencies of cases and controls were used to calculate the OR and the 95% CI. Multivariate logistic regression was used to adjust for potential confounding factors. When comparing severe sepsis group to sepsis group, we entered the genotypes or alleles in the multivariate models controlling for the confounding variables including age, gender, history of diseases, source of infection, APACHE II and SOFA scores. When comparing severe sepsis patients to healthy controls, age and gender were included in the multivariate models. The Bonferroni method was used to correct for multiple comparisons where applicable. The power analysis was performed using the Genetic Power Calculator web tool [45]. A two tailed P-value of <0.05 was considered statistically significant, whereas a value of corrected P<(0.05/number of tests) was considered significant after Bonferroni correction. Continuous variables were described as either a mean ± standard deviation, or as a median with interquartile range. TNF-α serum levels between individuals with different rs1800629 genotypes (AA+GA vs. GG) were compared by Student's *t*-test. To determine whether an association with rs1800629 genotypes might depend on other potential confounding factors for TNF-α serum levels, we investigated the association of rs1800629 genotypes by adding the polymorphisms to a linear regression model controlling for age, gender and APACHE II scores. The software used for statistical calculations was SPSS 15.0 (SPSS Inc., Chicago, IL, USA) unless specified.

## Supporting Information

Table S1 **Primers and PCR protocols for six SNPs in** ***TNF*** **and** ***LTA*** **.**
(DOC)

Table S2 **The primers of SNPs in** ***TNFRSF1A*** **and** ***TNFRSF1B*** **.**
(DOC)
