# Supplementary material for: Genetic Variation in the TNF Gene Is Associated with Susceptibility to Severe Sepsis, but Not with Mortality
Source: PLoS One. 2012 Sep 27;7(9):e46113. doi: 10.1371/journal.pone.0046113 (PMC3459853; doi:10.1371/journal.pone.0046113)
Supplement: Table S1 — Primers and PCR protocols for six SNPs in TNF and LTA . (DOC) [file pone.0046113.s001.doc]

**Table S1. Primers and PCR protocols for six SNPs in *TNF and LTA***

| Gene | Target Primer | Sequence (5'→3') | PCR protocol |
| --- | --- | --- | --- |
| TNF | rs1800629 |  | 95°C for 5 min; 35 cycles at 94°C for 30 s, 57°C for 40 s, |
|  | Forward | AACACAGCTTTTCCCTCCAA | and 72°C for 45 s; followed by 72°C for 10 min |
|  | Reverse | TAGCTGGTCCTCTGCTGTCC |  |
|  | rs361525 |  | 95°C for 5 min; 35 cycles at 94°C for 30 s, 57°C for 40 s, |
|  | Forward | AACACAGCTTTTCCCTCCAA | and 72°C for 45 s; followed by 72°C for 10 min |
|  | Reverse | TAGCTGGTCCTCTGCTGTCC |  |
|  | rs1799724 |  | 95°C for 5 min; 35 cycles at 94°C for 30 s, 57°C for 40 s, |
|  | Forward | CCTCGATGAAGCCCAATAAA | and 72°C for 45 s; followed by 72°C for 10 min |
|  | Reverse | ACTCTGGGGTCCCTGATTTT |  |
|  | rs1800630 |  | 95°C for 5 min; 35 cycles at 94°C for 30 s, 57°C for 40 s, |
|  | Forward | CCTCGATGAAGCCCAATAAA | and 72°C for 45 s; followed by 72°C for 10 min |
|  | Reverse | ACTCTGGGGTCCCTGATTTT |  |
|  | rs1799964 |  | 95°C for 5 min; 35 cycles at 94°C for 30 s, 57°C for 40 s, |
|  | Forward | CCTCGATGAAGCCCAATAAA | and 72°C for 45 s; followed by 72°C for 10 min |
|  | Reverse | ACTCTGGGGTCCCTGATTTT |  |
| LTA | rs909253 |  | 95°C for 5 min; 35 cycles at 94°C for 30 s, 57°C for 40 s, |
|  | Forward | CCACTGCCGCTTCCTCTAT | and 72°C for 45 s; followed by 72°C for 10 min |
|  | Reverse | AGGAGGAGGTGTAGGGTGGT |  |
