# Supplementary material for: Genetic Variation in the TNF Gene Is Associated with Susceptibility to Severe Sepsis, but Not with Mortality
Source: PLoS One. 2012 Sep 27;7(9):e46113. doi: 10.1371/journal.pone.0046113 (PMC3459853; doi:10.1371/journal.pone.0046113)
Supplement: Table S2 — The primers of SNPs in TNFRSF1A and TNFRSF1B . (DOC) [file pone.0046113.s002.doc]

***Table S2. The primers of SNPs in TNFRSF1A and TNFRSF1B***

| Gene | SNP | Oligo type-mode | Sequence |
| --- | --- | --- | --- |
| TNFRSF1A | rs767455 | rs767455_PCRU | AAGGTGCCTCGCCCACCA |
|  |  | rs767455_PCRL | AGTGAGAGGCCATAGCTGTCT |
|  |  | rs767455_SNPL | CGTGCCGCTCGTGATAGAATCCACCGTGCCTGACCTGCTGCTGCC |
| TNFRSF1A | rs4149570 | rs4149570_PCRU_1 | TTATCTATATCTCTCCCATCTGAAC |
|  |  | rs4149570_PCRU_2 | TTATTTATATCTCTCCCATCTGAAC |
|  |  | rs4149570_PCRL_1 | AATGTGTCTTGGACACACAAATG |
|  |  | rs4149570_PCRL_2 | AATGTGTCTTGGACACATAAATG |
|  |  | rs4149570_SNPU | GCGGTAGGTTCCCGACATATCAAATTGGAAAACAGATCCAGACAG |
| TNFRSF1B | rs1061622 | rs1061622_PCRU | TCCTCCTCCTCCAGCTGT |
|  |  | rs1061622_PCRL | TAAGTGTACTGCCCCTGGG |
|  |  | rs1061622_SNPU | ACGCACGTCCACGGTGATTTGTGGCCATCCCTGGGAATGCAAGCA |
| TNFRSF1B | rs3397 | rs3397_PCRU | CCAAGAGCAGAGGCAGCG |
|  |  | rs3397_PCRL_1 | ATGCCCCGAACATCCATG |
|  |  | rs3397_PCRL_2 | ATGCCCCGAACGTCCATG |
|  |  | rs3397_SNPL | GCGGTAGGTTCCCGACATATCCAGCCAGCCTTCCGAGAGGGACAC |

SNP, single nucleotide polymorphism
